# Identify Tcea3 as a novel anti-cardiomyocyte hypertrophy gene involved in fatty acid oxidation and oxidative stress

**DOI:** 10.3389/fcvm.2023.1137429

**Published:** 2023-06-19

**Authors:** Yingying Guo, Xian-feng Cen, Dan Li, Hong-liang Qiu, Ya-jie Chen, Meng Zhang, Si-hui Huang, Hao Xia, Man Xu

**Affiliations:** ^1^ Department of Cardiology, Renmin Hospital of Wuhan University, Wuhan, China; ^2^ Hubei Key Laboratory of Metabolic and Chronic Diseases, Renmin Hospital of Wuhan University, Wuhan, China; ^3^ Cardiovascular Research Institute, Wuhan University, Wuhan, China

**Keywords:** heart failure, bioinformatics, Tcea3, transcriptional regulator, fatty acid oxidation

## Abstract

**Background:**

Chronic pressure overload triggers pathological cardiac hypertrophy that eventually leads to heart failure. Effective biomarkers and therapeutic targets for heart failure remain to be defined. The aim of this study is to identify key genes associated with pathological cardiac hypertrophy by combining bioinformatics analyses with molecular biology experiments.

**Methods:**

Comprehensive bioinformatics tools were used to screen genes related to pressure overload-induced cardiac hypertrophy. We identified differentially expressed genes (DEGs) by overlapping three Gene Expression Omnibus (GEO) datasets (GSE5500, GSE1621, and GSE36074). Correlation analysis and BioGPS online tool were used to detect the genes of interest. A mouse model of cardiac remodeling induced by transverse aortic constriction (TAC) was established to verify the expression of the interest gene during cardiac remodeling by RT-PCR and western blot. By using RNA interference technology, the effect of transcription elongation factor A3 (Tcea3) silencing on PE-induced hypertrophy of neonatal rat ventricular myocytes (NRVMs) was detected. Next, gene set enrichment analysis (GSEA) and the online tool ARCHS4 were used to predict the possible signaling pathways, and the fatty acid oxidation relevant pathways were enriched and then verified in NRVMs. Furthermore, the changes of long-chain fatty acid respiration in NRVMs were detected using the Seahorse XFe24 Analyzer. Finally, MitoSOX staining was used to detect the effect of Tcea3 on mitochondrial oxidative stress, and the contents of NADP(H) and GSH/GSSG were detected by relevant kits.

**Results:**

A total of 95 DEGs were identified and Tcea3 was negatively correlated with Nppa, Nppb and Myh7. The expression level of Tcea3 was downregulated during cardiac remodeling both *in vivo* and *in vitro*. Knockdown of Tcea3 aggravated cardiomyocyte hypertrophy induced by PE in NRVMs. GSEA and online tool ARCHS4 predict Tcea3 involved in fatty acid oxidation (FAO). Subsequently, RT-PCR results showed that knockdown of Tcea3 up-regulated Ces1d and Pla2g5 mRNA expression levels. In PE induced cardiomyocyte hypertrophy, Tcea3 silencing results in decreased fatty acid utilization, decreased ATP synthesis and increased mitochondrial oxidative stress.

**Conclusion:**

Our study identifies Tcea3 as a novel anti-cardiac remodeling target by regulating FAO and governing mitochondrial oxidative stress.

## Introduction

Heart failure is a rapidly growing public health concern and a leading cause of mortality. Initially, cardiac hypertrophy develops as an adaptive response to persistent pathological stimuli, but it often progresses to fibrosis, dysfunction, and ultimately heart failure. Despite numerous studies on the mechanisms of heart failure, clinical outcomes remain suboptimal and the mortality rate is still high. Therefore, identification of new therapeutic targets is urgently required.

Transcription factors are an important type of gene transcription regulatory element. In addition to the translation and transcriptional initiation steps, transcriptional prolongation involving RNA polymerase (Pol) II is now considered to be a key step in controlling full-length mRNA production (1). It was found that some transcription enlongation factors such as positive transcriptional extension factor (P-TEF) B plays a key role in myocardial hypertrophy response to pressure overload (2). The paper pointed out that P-TEFB appears to be dynamically divided between the active state and the inactive state, indicating that its transcriptional control can be regulated (3). The three genes of vertebrate transcriptional extension factor TFIIS include Tcea1, 2,3. Among them, Tcea3 expression is restricted by tissues and is known to be highly expressed in intestine, heart, testis, kidney and skeletal muscle (4). Previous studies have shown that Tcea3 is associated with skeletal muscle cell differentiation (5) and that Tcea3 binds to TGF- RI, thereby inducing SMAD- independent and JNK- dependent apoptotic pathways to regulate angiogenesis (6). Other studies have shown that Tcea3 is a necessary cofactor for MRF driver gene expression during myogenesis (7). Since TFIIS coordinate the heart's response to hypertrophic stimuli with numerous gene expression in adults, cardiac Tcea3 may be a promising therapeutic target. However, there is no report on the research of Tcea3 in heart disease.

FAO provides the majority of energy in healthy adult cardiomyocytes, but changes occur during pathological cardiac remodeling (8). The disorder of fatty acid metabolism in heart is considered to be a consistent feature of heart failure (9). Indeed, several genes concerned in fatty acid *β*-oxidation are shown to possess a vital role within the pathological progression of cardiac remodeling. Inhibition of fatty acid oxidation by CPT1B deficiency had been shown leading to myocardial lipid accumulation and exacerbate cardiac pathological progress (10). Studies have suggested that levels of some lipid metabolism-related proteins are associated with heart failure events. For example, plasma activity of sPLA2 was increased in non-ischemic heart failure patients compared to controls (11) and Lp-PLA2 activity is independently associated with incident heart failure (12, 13). Mitochondria are the main site of fatty acid oxidation, which is accompanied by the production of mitochondrial ROS. As HF progresses, FA oxidation and mitochondrial oxidative activity decrease, leading to a significant decrease in cardiac ATP levels and compensatory upregulation of glucose uptake and glycolysis, but this upregulation is insufficient to compensate for the decrease in ATP production. Increased mitochondrial ROS production and ROS-mediated damage leading to HF progression when they overwhelm the cellular antioxidant defense system (14). Nonetheless, due to the complexity of the regulation mechanism between fatty acid oxidation and mitochondrial ROS has not been fully elucidated.

In this study, we employed a range of bioinformatics tools to identify Tcea3 as a differentially expressed gene, and predicted its involvement in fatty acid oxidation during cardiac hypertrophy. Since cardiac hypertrophy is associated with substrate conversion from fatty acids to glucose, we hypothesized that decreased Tcea3 leads to decreased fatty acid oxidation and increased mitochondrial oxidative stress, ultimately leading to further exacerbation of cardiac hypertrophy. We confirmed the differential expression of Tcea3 during cardiac remodeling both *in vivo* and *in vitro*. Consistent with the hypothesis, Tcea3 silencing aggravated PE induced cardiomyocyte hypertrophy, inhibited the expression of Ces1d and Pla2g5, impaired fatty acid oxidation, decreased ATP synthesis, and increased mitochondrial oxidative stress. Collectively, we reported a new target for the treatment of cardiomyocyte remodeling in NRVMs stimulated with PE and points out for the first time that Tcea3 participate in FAO and highlight the role of Tcea3 in oxidative stress.

## Methods

### Differentially expressed genes (DEGs)

Gene expression profiles with series number GSE5500, GSE1621, and GSE36074 were downloaded from the Expression Omnibus Gene (GEO) database. Limma of R/bio-conductor was used for screening of the DEGs (settings: *P* < 0.05, log2|fold change |>0.5), and differentially expressed genes were identified. The volcano plots and heatmaps were created by R software “pheatmap” package (version 1.0.12; https://cran.r-project.org/web/packages/pheatmap/). The basic information of the datasets is shown in Supplementary Excel file.

### Gene set enrichment analysis (GSEA)

GSEA software V2.1.0 was used to identify the pathway associated with Tcea3 expression, and the expression matrix was 32,619(gene) × 14(sample). Predefined gene set “c2.all.v4.0.symbols” was selected. Enrichment results satisfying a nominal *P*-value < 0.05 and a false discovery rate (FDR) < 0.25 were considered statistically significant.

### Human heart samples

The hearts were obtained from the left ventricles of patients with DCM and non-cardiac deceased donors, which were all not suitable for transplantation. All procedures involving human samples were in accordance with the declaration of Helsinki, and informed consent was obtained from the families of all donors. The review committee of the People's Hospital of Wuhan University approved this study. The source of these donors has previously been identified (15, 16). The demographics of human heart samples are shown in Supplementary document Table S4.

### Animals and animal models

We obtained all of our male C57BL/6 mice from the Institute of Laboratory Animal Science, Chinese Academy of Medical Sciences, at 8–10 weeks of age and 23.5–27.5 g of body weight. All animal care and experimental procedures are approved by the Animal Care and Use Committee of Wuhan University People's Hospital (Wuhan, China). Transverse aortic constriction (TAC) was conducted to achieve overload-induced cardiac hypertrophy and heart failure in mice as previously described (17). Briefly, A 27G needle was used to ligate the aorta of male mice after they were intraperitoneally injected with 3% pentobarbital sodium at 40 mg/kg. The transverse aorta was not ligated in sham-operated mice. We dislocated and executed mice after 4 weeks of sham or TAC surgery, and removed heart tissue for further analysis.

### Neonatal rat ventricular myocytes (NRVMs) isolation

Briefly, ventricular cardiomyocytes were isolated from the left ventricles of 1–2-day-old Sprague Dawley rats and digested with Type 2 collagenase (0.5 mg/ml) (Worthington Biochemical Corp, NJ) and pancreatin (0.6 mg/ml) (Sigma). The Isolated cardiomyocytes were re-suspended in DMEM containing 10%FBS and precultured for 24 h. Then Cells were incubated with 12%FBS containing Phenylephrine (100uM; ab120761; abcam) for 48 h to stimulate cardiomyocyte hypertrophy.

### RNA interference

The sequences for rTcea3 siRNA were as follows: Sense sequence: 5′ GUGCAAGAAGAAGAAUUGUACCUAT 3′, antisense sequence:

5′ AUAGGUACAAUUCUUCUUCUUGCACUU 3′. The scrambled and Tcea3 siRNAs were transfected into NRVMs with the RNAiMAX (Invitrogen, 13778150) according to the manual. RNAi duplex-LipofectamineTM RNAiMAX complexes (Contains 5 µl of RNAiMAX) should be added to each well of a 6-well culture vessel. This gives a final volume of 3 ml and a final RNA concentration of 20 nM. Twelve hours after transfection, PE was added to induce cardiomyocyte hypertrophy. Cells were harvested 48 h after transfection and the levels of mRNA were assessed by RT-PCR.

### Western blot and quantitative real-time PCR

After the proteins were extracted from frozen hearts or cultures, they were electrophoresed on 10% sodium dodecyl sulfate-polyacrylamide gels and subsequently transferred onto polyvinylidene difluoride membranes. A secondary antibody incubation at room temperature for 60 min followed the overnight at 4°C incubation with the primary antibodies against Tcea3(Santa Cruz Biotechnology, sc−365894, 1:1000 dilution). Total RNA was extracted from frozen heart tissue and primary cultured cardiomyocytes, using Trizol Reagent (Invitrogen). Reverse transcription (RT) was carried out with the First Stand cDNA Synthesis Kit (Thermo Scientific) according to the manufacturer's protocol. After the cDNA was obtained and diluted to 80ul, SYBR Green chemistry with GAPDH or Tcea3 primers were used to amplify. The primer sequences are shown in Supplementary document Table S5.

### Measurement of long-chain fatty acid respiration in NRVMs

Oxygen consumption rate (OCR) were measured using a Seahorse Bioscience XF24 instrument (Seahorse Bioscience, North Billerica, MA). A density of 80,000 isolated NRVMs per well was seeded into Seahorse XF cell culture microplates. Using Seahorse XF calibration solution, a sensor probe plate was hydrated overnight at 37 °C in CO2-free conditions. Preparation of the mitochondrial stress test kit (Cat.103015–100; Agilent, CA, USA) and the assay solutions was done in advance. After washing the cells with the FAO assay solution supplemented with 2.5 mM glucose, 0.5 mM carnitine, 500 μl of the assay solution was added to the cells. Following this, we incubated the sample at 37°C for 1 h in a CO_2_-free incubator. The sensor probe plate was preloaded with oligomycin, FCCP, and rotenone/antimycin. Finally, the changes of long-chain fatty acid respiration in NRVMs were detected using the Seahorse XFe24 Analyzer (Agilent).

### Determination of superoxide levels

The treated cells were incubated with 5 μM MitoSox red mitochondrial superoxide indicator (M36008; Invitrogen) at 37 °C in the absence of light for 30 min to measure the level of superoxide in the mitochondria. Following washing, the cells were observed using a conventional fluorescence microscope(Olympus, Japan).

### Statistical analysis

Data are expressed as the mean ± standard error of the mean of at least three independent experiments. In order to determine statistical significance, the results were compared using Student's two-tailed *t* test. All statistical analyses were performed using GraphPad Prism 5.0 software. At the 95% confidence level, a *P* value of < 0.05 is considered to indicate a statistically significant difference.

## Results

### Identification of DEGs

**Figure 1:**
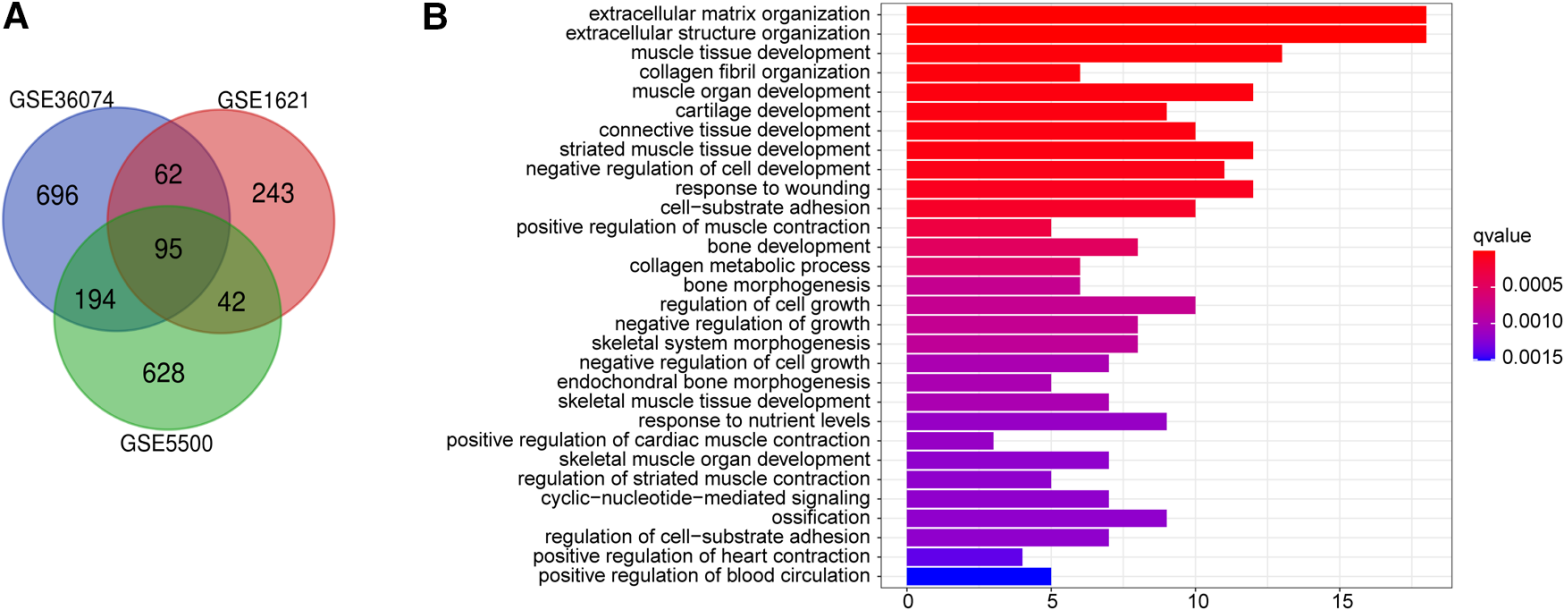
Function annotations for deregulated genes in cardiac remodeling. (**A**) Venn diagram of the 17 downregulated and 78 upregulated DEGs based on the three GEO datasets. (**B**) The top 30 enriched gene ontology (GO) biological process (BP) terms BP.

Transaortic constriction (TAC) is performed by ligating the aortic arch of the heart, and the long-term high afterload of the heart causes adaptive changes in the myocardium, leading to cardiac remodeling. To detect the vital genes involved in cardiac remodeling, we employ three gene expression profiles including GSE5500, GSE1621 and GSE36074, both of which were from TAC-induced cardiac hypertrophic mouse and control mouse heart. 95 DEGs in total were identified according to screening criteria (*P* < 0.05, |fold change| > 0.5). The volcano plots and heatmaps of the differentially expressed genes are displayed in Supplementary Figures S1A-C. Next, using the Venn website, 78 up-regulated genes and 17 down-regulated genes were obtained from the three profile datasets (Figure 1A), detail of which was shown in Supplementary Table S1.

To acquire a deeper understanding of DEGs, enrichment of the functions and pathways were analyzed by employing R language “ggplot2″ “ClusterProfiler” and “enrichplot” package to visualize the GO annotation result data. Adj. *P* < 0.05 was regarded as statistically significant. Supplementary Table S2 lists the top 30 enriched GO biological process (BP) terms. GO biological process analysis revealed that the top three significantly enriched terms of these 95 DEGs were extracellular matrix organization, extracellular structure organization, muscle tissue development (Figure 1B).

### Identifying Tcea3 as the gene of interest

**Figure 2:**
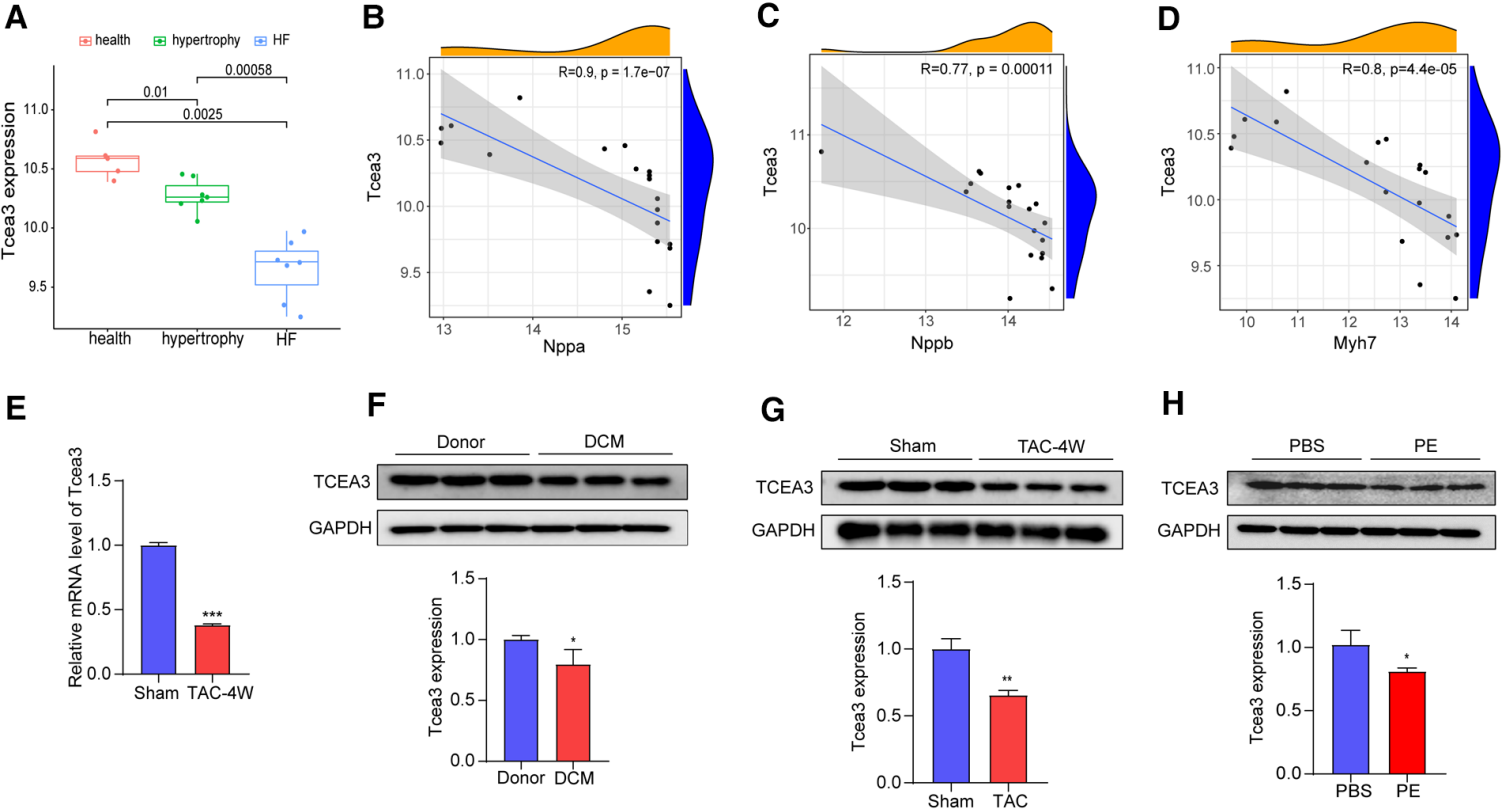
Validation of the Tcea3 expression. (**A**) The box plot is based on 7 hypertrophy samples (marked in green), 7 HF samples (marked in blue) and 5 normal samples (marked in red) that revealed expression of Tcea3 in the left ventricle (wilcox.test) in GSE36074. Cardiac hypertrophy was defined as lung weight/tibia length ≤ 10.5 mg/mm and LAD ≤ 2.0 mm and HF was defined as lung weight/tibia length ≥ 15 mg/mm and LAD ≥ 2.4 mm. LAD, left atrial diameter. (**B-D**) Spearman correlation analysis of Tcea3 with the Nppa, Nppb and Myh7, respectively. (**E**) Relative mRNA expression level of Tcea3 in Sham or TAC group (*n* = 3). (**F**) Western blot and quantitation of Tcea3 protein level in the hearts of healthy individuals and DCM patients (*n* = 3). (**G**)Western blot and quantitation of Tcea3 protein level in the hearts of mouse subjected to sham or TAC surgery at 4 weeks (*n* = 3). (**H**) Western blot and quantitation of Tcea3 protein level in phenylephrine (PE) induced cardiomyocyte hypertrophy(*n* = 3). Data are presented as mean ± SEM. *p < 0.05; **p < 0.01.

Heart failure is the end-stage change of myocardial remodeling and is one of the leading causes of mortality. Exploring genes that play an important role in the transition from hypertrophy to dysfunction will help to find genes that play key roles in HF progression. Since the hearts in the GSE36074 study were divided into HF group and nonHF group based on lung weight and left atrial diameter (LAD), we selected the GSE36074 dataset to further identify the key genes among these 95 genes closely related with the transition from cardiac hypertrophy to heart failure. As a result, a total of 25 genes were significantly associated with heart failure progression (*p* value < 0.001) (Supplementary Table S3). Next, ten genes (Ces1d, Tcea3, Postn, Mfap5, Col5a2, Cmtm8, Apbb1, Serpinf1, Fgl2 and Bgn) were further identified specific expressed in heart tissue by using BioGPS (the tissue-specific expression level was >10 times the median). Among these genes, Tcea3 is one of transcriptional extension factor TFIIS which is known to be highly expressed in heart. Interestingly, to our knowledge, the role of Tcea3 in heart disease has not been reported yet. Box-plot revealing that the expression levels of Tcea3 is significantly different between failure heart, nonfailure heart and sham heart in the GSE36074 dataset (Figure 2A). We further conduct correlation analysis between Tcea3 and the cardiac hypertrophy markers (18). As shown in Figures 2B–D, Tcea3 negatively related with ANP, BNP and Myh7. Therefore, Tcea3 was selected as the interest gene for further study.

To validate Tcea3 expression levels during cardiac hypertrophy, the heart samples from healthy individuals and DCM patients were used. As shown in Figure 2F, compared with the donor group, the expressions of Tcea3 were downregulated, which was consistent with the results of bioinformatics analysis. Furthermore, we subjected 8-week male WT mice to pressure overload by TAC surgery (Supplementary Figures S2A-I). As expected, the mRNA expression level of ANP and BNP were higher in TAC hearts than in sham hearts (Supplementary Figures S2E,F). Moreover, TAC mice developed more interstitial fibrosis and cardiac myocyte enlargement compared to sham mice (Supplementary Figure S2I). The above results indicate that the TAC-induced cardiac hypertrophy mouse model was successfully constructed. Next, we detect the expression levels of Tcea3 in this mouse model by RT-PCR and the results are shown in Figure 2E. Consistent with the mRNA changes, the protein level of Tcea3 also decreased after TAC surgery compared with sham-operated group (Figure 2G). In addition, we isolated NRVMs from neonatal rats and used phenylephrine (PE) to induce cardiomyocyte hypertrophy. Similarly, compared with the control group, and the expression level of Tcea3 in PE induced group also decreased (Figure 2H).

### Knockdown of Tcea3 in NRVMs aggravates cardiomyocyte hypertrophy

**Figure 3:**
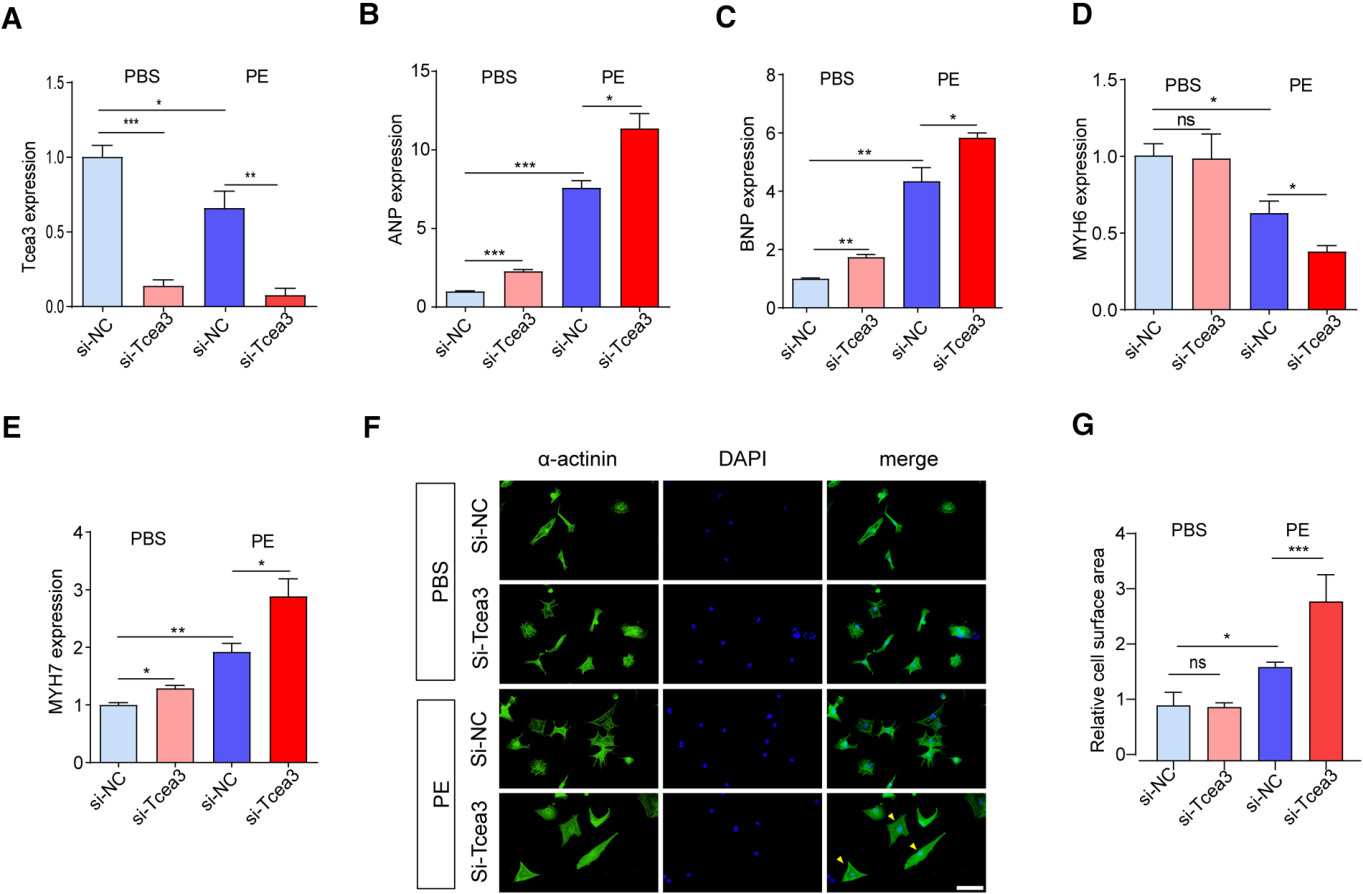
Silencing of Tcea3 in NRVMs aggravates cardiomyocyte hypertrophy. (**A**) Relative mRNA levels of Tcea3 in NRVMs. (**B-E**) Relative mRNA levels of cardiac hypertrophy marker genes in NRVMs transfected with control siRNA or Tcea3 siRNA followed by treatment with phenylephrine or PBS (20 μM) for 24 h (*n* = 3). (**E,F**) Representative *α*-actinin-stained immunofluorescence images (**E**) and Quantitative results of the cell surface area (**B**) of NRVMs (*n* = 3). The arrows show representative NRVMs with hypertrophy. Scale bar, 50 μm. Data are presented as mean ± SEM. *P < 0.05, ** *P* < 0.01, ***P < 0.001. PE, phenylephrine; DAPI, 4′,6-Diamidino-2′-phenylindole; ANP, atrial natriuretic peptide; BNP, b-type natriuretic peptide; MYH6, Myosin Heavy Chain 6; MYH7, Myosin Heavy Chain 7.

The above results indicate Tcea3 associated with the progression of heart failure and confirm the decreased expression of Tcea3 in cardiac hypertrophy, we speculate Tcea3 is likely to play an important role in the progression of heart failure. To verify this hypothesis, RNA interference was used to silence the expression of Tcea3 in NRVMs (Figure 3A). Decrease of Tcea3 protein level induced by RNA interference is confirmed (Supplementary Figure S3). Following PE stimulation, RT-PCR analysis revealed that Tcea3 knockout significantly upregulated the expressions of ANP, BNP, and MYH7, while significantly downregulating MYH6 expression, as compared to the Si-NC group (Figures 3B–E). Moreover, compared with the control siRNA group, knockdown of Tcea3 by siRNA result in increased cell area by *α*-actinin staining (Figures 3F,G). The above experimental evidence indicates that knockdown of Tcea3 by siRNA in NRVMs aggravate cardiomyocyte hypertrophy induced by PE.

### Tcea3 is involved in the lipid metabolism signaling pathways during cardiac remodeling

**Figure 4:**
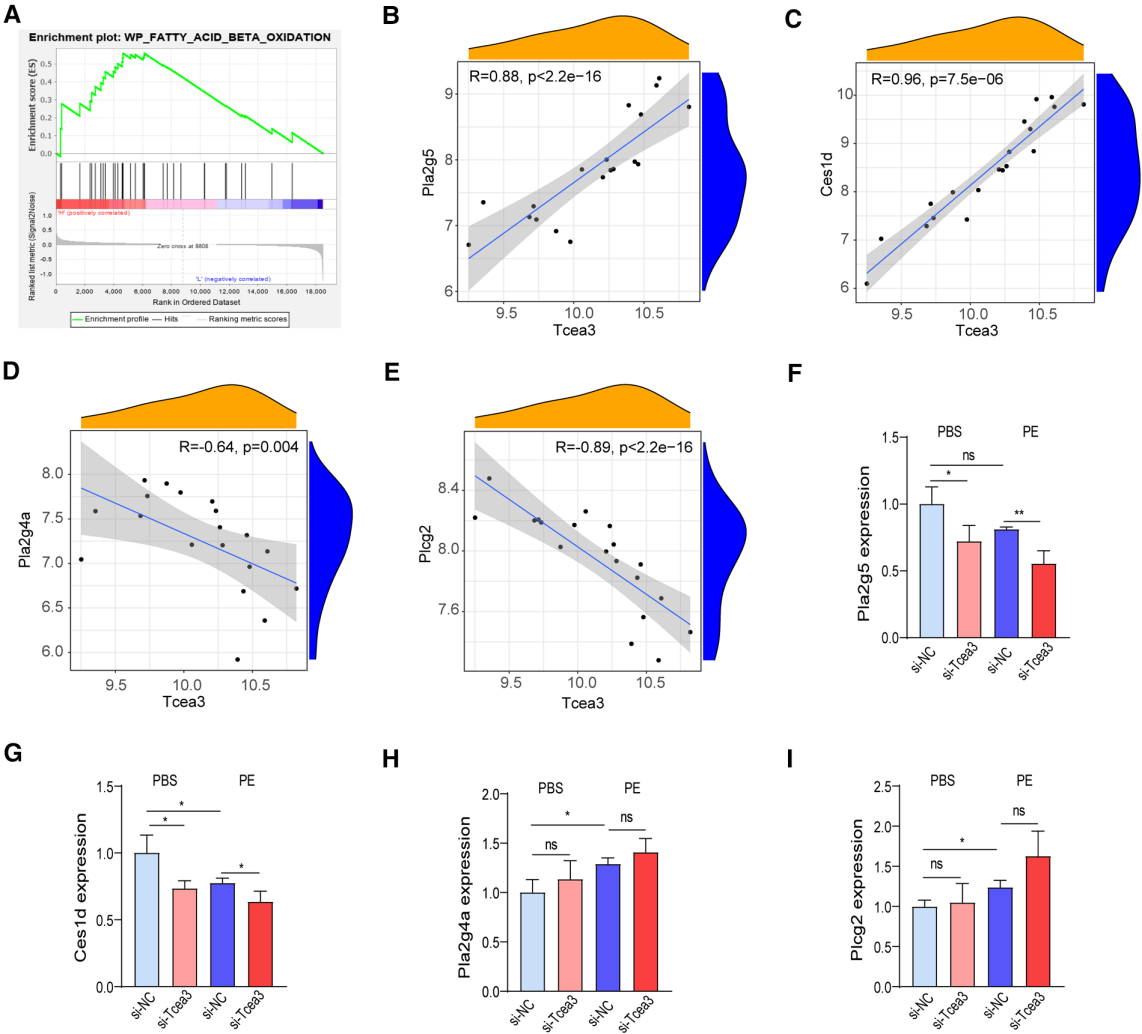
Tcea3 participates in lipid metabolism signaling pathways. (**A**) GSEA reveals fatty acid beta oxidation signaling pathway were enriched in the low-Tcea3 expression group. Top panels indicate the enrichment scores for each gene. Bottom panels show the ranking metrics of each gene. Y-axis: ranking metric values; X-axis: ranks for all genes. NES: normalized enrichment score. (**B-E**) Spearman correlation analysis of Tcea3 with the expression of Pla2g5, Ces1d, Pla2g4a, and Plcg2, respectively. (**F-I**) RT-PCR results of the four genes expression level in NRVMs transfected with control siRNA or Tcea3 siRNA followed by treatment with phenylephrine (20 μM) for 24 h. (*n* = 3). Data are presented as mean ± SEM. *P < 0.05, ** *P* < 0.01.

To further understand the biological function of Tcea3 in the process of cardiac remodeling, we performed GSEA analysis based on the expression level of Tcea3 to determine the molecular mechanism of Tcea3 affecting the progression of heart failure. According to the median expression level of Tcea3, the 14 TAC samples download from GSE36074 dataset were divided into Tcea3-low (*n* = 7) and Tcea3-high (*n* = 7) groups. GSEA analysis revealed a significant association between Tcea3 and mitochondrial biogenesis, MAPK cascade, fatty acid beta oxidation (Figure 4A) and mitochondrial calcium ion transport. On the basis of the top 10 predicted pathways (KEGG) of Tcea3 predicted by Online tool ARCHS4 (https://maayanlab.cloud/archs4/) and GSEA enrichment results, we finally speculate that Tcea3 may be related to fatty acid beta oxidation.

Fatty acids are the main energy substrate of the heart and provide necessary cofactors for mitochondrial oxidative phosphorylation. Data to date suggest that fatty acid oxidation plays an important role in the progression of heart disease and that increasing myocardial fatty acid oxidation helps improve cardiac function in heart failure (19, 20). Interestingly, among the 95 differentially expressed genes, Ces1d, Plcg2, Pla2g4a and Pla2g5 were also involved in the lipid metabolism. Therefore, we hypothesized that Tcea3, as a transcription lengthening factor, might regulate the transcription of the 4 genes. Then correlation analysis conducted between Tcea3 and these 4 genes respectively. The results showed that the mRNA level of Tcea3 was positively correlated with the mRNA level of Pla2g5 and Ces1d (Figures 4B,C), while negatively correlated with the mRNA level of Pla2g4a and Plcg2 (Figures 4D,E). Finally, we examined the mRNA levels of these four genes in PE-induced cardiac hypertrophy using RT-PCR. Compared with the PBS-treated cardiomyocytes, the mRNA level of Ces1d was down-regulated, Pla2g4a and Plcg2 were up-regulated (Figures 4G-I), while Pla2g5 was not significantly altered in PE-treated cardiomyocytes (Figure 4F).

**Figure 5:**
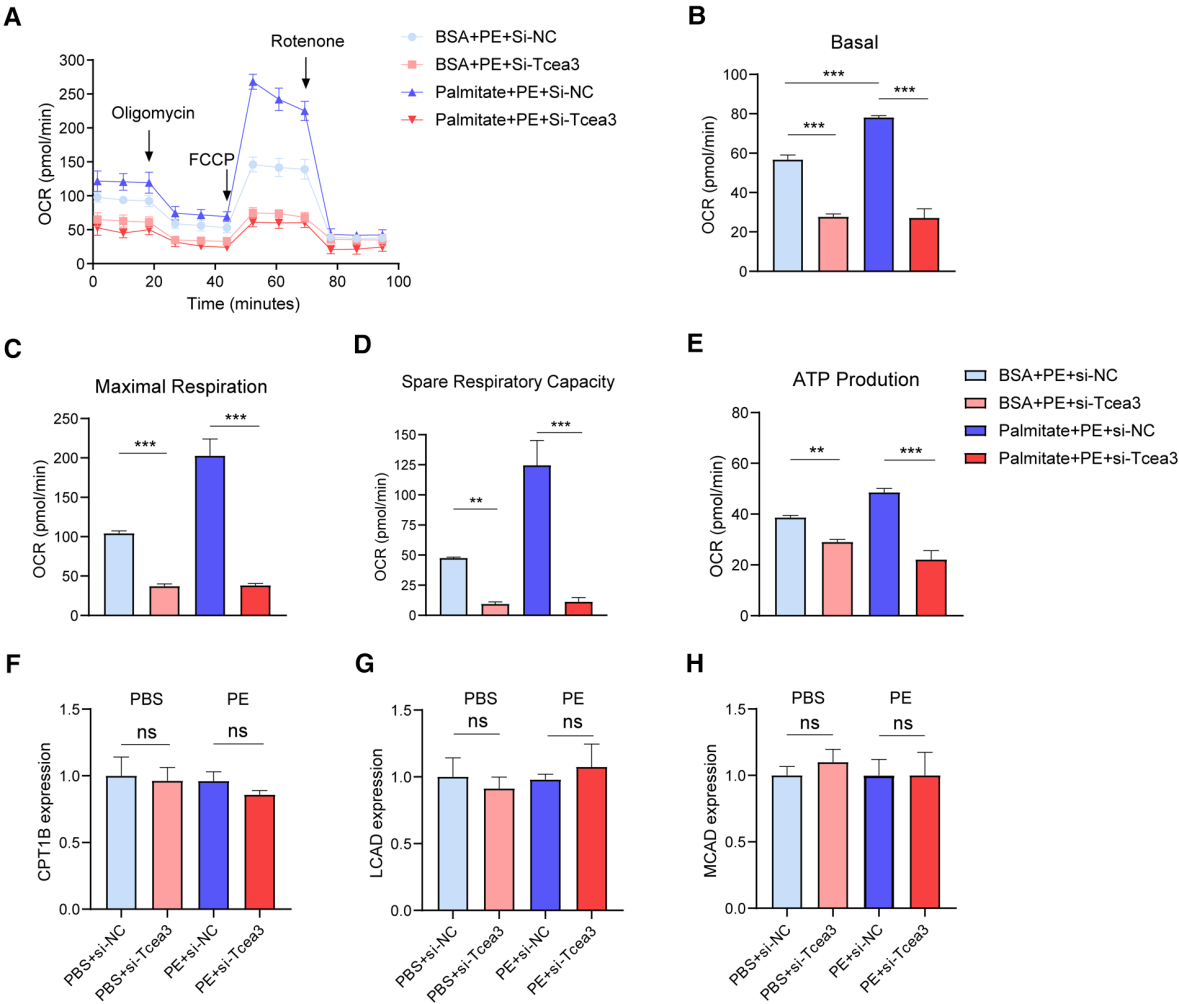
The impact of Tcea3 on fatty acid oxidation in NRVMs. (**A**) Oxygen consumption rate (OCR) in NRVMs transfected with control siRNA or Tcea3 siRNA followed by treatment with phenylephrine (20 μM) for 24 h. OCR was real-time detected in living NRVMs by Seahorse XFe24 Analyzer. (**B-E**) Quantification of basal respiration, maximal respiration, spare respiration capacity and ATP production. (**F-H**) Relative mRNA levels of genes involved in FAO in NRVMs (*n* = 3). Data are presented as mean ± SEM. *P < 0.05, ** *P* < 0.01, ***P < 0.001.

To determine the potential role of Tcea3 in cardiomyocyte long-chain fatty acid β-oxidation, Seahorse XFe24 extracellular flux analyzer was used to evaluation long-chain fatty acid respiration. Compared with BSA-treated cells, oxygen consumption rate (OCR) of NRVMs treated with palmitate was significantly upregulated. In the presence of palmitic acid, Tcea3 knockdown in NRVMs resulted in a significant proportional decrease in basal and maximum respiration rate. Spare respiratory capacity and ATP production derived from oxidative phosphorylation was also significantly impaired in response to Tcea3 silencing (Figures 5A-E). Since CPT1B, MCAD and LCAD are widely recognized as the key enzyme involved in mitochondria β-oxidation (21), we also detect their gene expression levels. RT-PCR results showed that both CPT1B, LCAD and MCAD mRNA level were not affected after Tcea3 knockdown (Figures 5F–H). These results suggest that Tcea3 can regulate fatty acid β-oxidation in cardiomyocytes, but not through the regulation of CPT1B, LCAD, and MCAD expression.

### Tcea3 silencing exacerbates oxidative stress

The relationship between heart hypertrophy, fatty acid oxidation and mitochondrial ROS has been studied for a long time. We have shown that Tcea3 governs the expression of Ces1d and Pla2g5 involved in FAO. In addition, the results above show that that knockdown of Tcea3 from cardiomyocytes exacerbates cardiomyocyte hypertrophy. Consider the close link between fatty acid oxidation and mitochondrial ROS during cardiac hypertrophy, We next asked whether reduction in oxidative stress is a mechanism underlying Tcea3-mediated protection of cardiac function.

**Figure 6:**
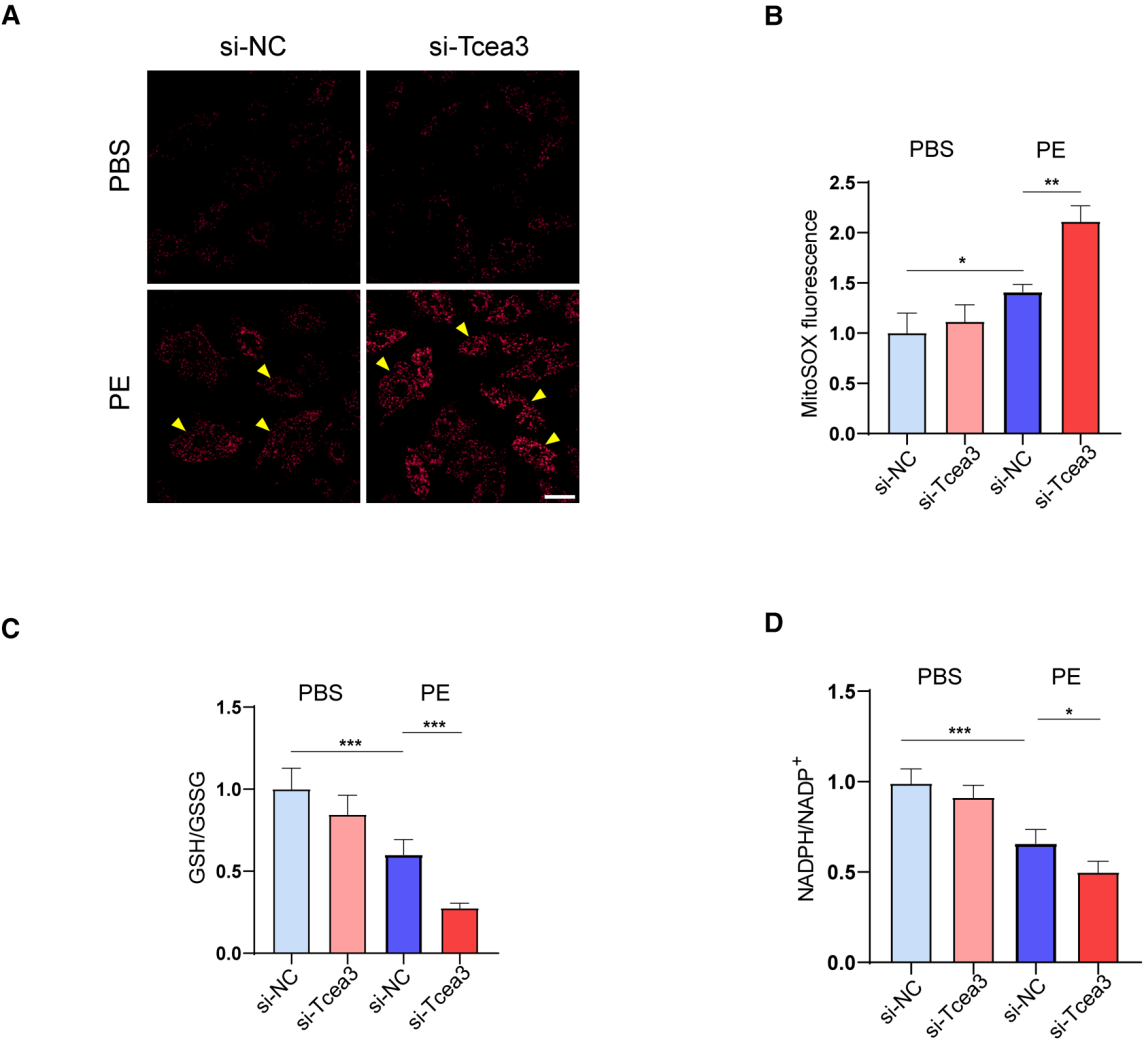
Effects of Tcea3 on oxidative stress in NRVMs. (**A,B**) Representative MitoSOX Red fluorescence images of NRVMs and the quantification of MitoSOX Red fluorescence intensity (*n* = 3). The arrows show representative NRVMs with oxidative stress. Scale bar, 20 μm. (**C**) Levels of GSH/GSSG in NRVMs of the four treatment groups (*n* = 5). (**D**)The level of NADPH/NADP^+^ in NRVMs transfected with control siRNA or Tcea3 siRNA and treated with phenylephrine (20 μM) or PBS for 24 h (*n* = 5). Data are presented as mean ± SEM. *P < 0.05, **P < 0.01. PE, phenylephrine; GSH, glutathione; GSSG, glutathione disulfide; NADP^+^, Nicotinamide adenine dinucleotide phosphate.

Firstly, we detected the level of mitochondrial ROS in isolated cardiomyocytes. The immunofluorescence staining showed that PE stimulated the increase of mitochondrial ROS compared with the normal control group, while knockdown of Tcea3 further increased MitoSOX fluorescence signal (Figures 6A,B). Along this line, we further found that Tcea3 silencing resulted in a decrease in the GSH/GSSG ratio in PE induced cardiomyocyte hypertrophy (Figure 6C). NADPH is used to maintain glutathione and thioredoxin in a reduced state and is controlled by a variety of metabolic pathways and enzymes. As shown in (Figure 6D), knockdown of Tcea3 in NRVMs reduced NADPH production and the ratio of NADPH to NADP^+^ in PE stimulated group. These findings collectively suggest that Tcea3 is essential for controlling oxidative stress.

## Discussion

In recent years, with the emergence and rapid development of gene chip (microarray) and high-throughput sequencing technology, it provides a valuable method for the prevention, early diagnosis, treatment and prognosis of diseases including HF. The present study points out the anti-cardiomyocyte hypertrophy effect of Tcea3, which is related to fatty acid metabolism and oxidative stress for the first time.

Firstly, 78 up-regulated genes and 17 down-regulated differential expressed genes were obtained through overlap analysis of three datasets. Among these genes, studies have confirmed that some genes are related to myocardial hypertrophy. In addition to Nppb, Nppa, and Myh7, these differentially expressed genes also include some myocardial remodeling-related genes. For example, TGFB2, a multifunctional cytokine that can be involved in myocardial and vascular remodeling, has been proved to be up-regulated in peripheral blood mononuclear cells with cardiovascular structural changes in hypertensive patients, suggesting that TGFB2 monocyte production may play a role in hypertension (22). SERPINE-1, also known as plasminogen activator inhibitor type I (PAI-1) have been shown to promote Cardiomyocyte fibrosis by regulating TGFB2 signal pathway (23). These results indicate that the differentially expressed genes we identified through bioinformatics analysis have significant reference value.

In this study, we identified Tcea3 as a potential target gene for anti-myocardial remodeling through bioinformatics analysis. Subsequently, we confirmed that knockdown of Tcea3 inhibited PE-induced cardiomyocyte hypertrophy in NRVMs. We also detected downregulation of Tcea3 expression in dilated cardiomyopathy tissue from humans, indicating the potential clinical application of Tcea3 as an anti-myocardial remodeling agent, which, however, needs to be further confirmed. Mechanistically, by using GSEA single gene enrichment analysis and the ARCHS4 online website, we found that Tcea3 is related to fatty acid degradation. Abnormalities in fatty acid metabolism are widely present in myocardial remodeling, and targeting fatty acid metabolism has become a hot topic in anti-myocardial remodeling therapy (24–26). Interestingly, among the differentially expressed genes we identified in this screening, Ces1d, Plcg2, Pla2g4a, and Pla2g5 are all involved in lipid metabolism. Our experiments on cardiomyocytes showed that the mRNA level of Ces1d was down-regulated in PE-treated cells after Tcea3 knockdown (Figure 4F), suggesting that Ces1d may be an effective target gene regulated by Tcea3. Ces1d is a carboxylesterase with broad activity in multiple tissues, involved in regulating systemic fatty acid and cholesterol metabolism (27, 28). The role of Ces1d in myocardial remodeling has not been reported yet, and whether the effect of Tcea3 on PE-induced cardiomyocyte hypertrophy depends on Ces1d remains to be further validated. In future studies, it is worth paying attention to whether the influence of Tcea3-mediated Ces1d abnormal expression on myocardial lipid metabolism is the result of local regulation of myocardial cell energy metabolism or the result of systemic multi-tissue and organ interaction. In this study, we also found that knockdown of Tcea3 in NRVMs stimulated with PE inhibited the utilization of fatty acids and reduced ATP production. It can be speculated that Tcea3 knockdown leads to abnormal myocardial energy metabolism, which may be an important reason for aggravating PE-induced cardiomyocyte hypertrophy. Our study suggests that Tcea3 may be an important member of the cardiac metabolism regulatory network and deserves attention in f uture studies on myocardial energy metabolism.

Recent studies have shown that the process of fatty acid oxidation is accompanied by the production of NADPH (29–31), which is an important antioxidant molecule that maintains glutathione and thioredoxin in the reduced state and is involved in the regulation of intracellular oxidative stress. We have therefore also focused on the effect of Tcea3 on oxidative stress in PE-induced cardiomyocytes. It was found that Tcea3 knockdown did affect mitochondrial oxidative stress, mainly by increasing mitochondrial ROS levels, decreasing the level of GSH/GSSG and NADPH/NADP+ ratio. However, the present study suggests a possible link between Tcea3 and NADPH, but further studies are needed to confirm whether NADPH is an intermediate bridge linking fatty acid oxidation and oxidative stress.

There are also some limitations in our analysis. The small sample size may make our candidate genes inconsistent with the real situation. In addition, the function of Tcea3 needs further verification of animal research. In conclusion, we identifies Tcea3 as a novel anti-cardiac remodeling target by regulating FAO and governing mitochondrial oxidative stress. We predict that the role of Tcea3 in myocardial lipid metabolism may provide new insights into the pathogenesis of cardiovascular diseases in clinical settings. The specific mechanism by which Tcea3 regulates myocardial fatty acid oxidation and lipid metabolism, as well as its crosstalk with oxidative stress, are directions for our future research.

## Data Availability

The datasets presented in this study can be found in online repositories. The names of the repository/repositories and accession number(s) can be found in the article/**Supplementary Material**.

## References

[1] Zhou Q Yik JHN. The yin and yang of P-TEFb regulation: implications for human immunodeficiency virus gene expression and global control of cell growth and differentiation. Microbiol Mol Biol Rev. (2006) 70(3):646–59. 10.1128/MMBR.00011-06 16959964 PMC1594588

[2] Sano M Abdellatif M Oh H Xie M Bagella L Giordano A Activation and function of cyclin T-Cdk9 (positive transcription elongation factor-b) in cardiac muscle-cell hypertrophy. Nat Med. (2002) 8(11):1310–7. 10.1038/nm778 12368904

[3] Espinoza-Derout J Wagner M Salciccioli L Lazar JM Bhaduri S Mascareno E Positive transcription elongation factor b activity in compensatory myocardial hypertrophy is regulated by cardiac lineage protein-1. Circ Res. (2009) 104(12):1347–54. 10.1161/CIRCRESAHA.108.191726 19443839 PMC2774227

[4] Labhart P Morgan GT. Identification of novel genes encoding transcription elongation factor TFIIS (TCEA) in vertebrates: conservation of three distinct TFIIS isoforms in frog, mouse, and human. Genomics. (1998) 52(3):278–88. 10.1006/geno.1998.5449 9790746

[5] Zhu Y Tong HL Li SF Yan YQ. Effect of TCEA3 on the differentiation of bovine skeletal muscle satellite cells. Biochem Biophys Res Commun. (2017) 484(4):827–32. 10.1016/j.bbrc.2017.01.182 28161635

[6] Cha Y Kim DK Hyun J Kim SJ Park KS. TCEA3 Binds to TGF-beta receptor I and induces smad-independent, JNK-dependent apoptosis in ovarian cancer cells. Cell Signal. (2013) 25(5):1245–51. 10.1016/j.cellsig.2013.01.016 23357533

[7] Kazim N Adhikari A Davie J. The transcription elongation factor TCEA3 promotes the activity of the myogenic regulatory factors. PLoS One. (2019) 14(6):e0217680. 10.1371/journal.pone.0217680 31158246 PMC6546274

[8] Wang C Yuan Y Wu J Zhao Y Gao X Chen Y Plin5 deficiency exacerbates pressure overload-induced cardiac hypertrophy and heart failure by enhancing myocardial fatty acid oxidation and oxidative stress. Free Radic Biol Med. (2019) 141:372–82. 10.1016/j.freeradbiomed.2019.07.006 31291602

[9] Stanley WC Recchia FA. Lipotoxicity and the development of heart failure: moving from mouse to man. Cell Metab. (2010) 12(6):555–6. 10.1016/j.cmet.2010.11.016 21109186 PMC3330240

[10] He L Kim T Long Q Liu J Wang P Zhou Y Carnitine palmitoyltransferase-1b deficiency aggravates pressure overload-induced cardiac hypertrophy caused by lipotoxicity. Circulation. (2012) 126(14):1705–16. 10.1161/CIRCULATIONAHA.111.075978 22932257 PMC3484985

[11] Varricchi G Loffredo S Bencivenga L Ferrara AL Gambino G Ferrara N Angiopoietins, vascular endothelial growth factors and secretory phospholipase A2 in ischemic and non-ischemic heart failure. J Clin Med. (2020) 9(6):1928. 10.3390/jcm9061928 32575548 PMC7356305

[12] van Vark LC Kardys I Bleumink GS Knetsch AM Deckers JW Hofman A Lipoprotein-associated phospholipase A2 activity and risk of heart failure: the rotterdam study. Eur Heart J. (2006) 27(19):2346–52. 10.1093/eurheartj/ehl230 16952920

[13] Suzuki T Solomon C Jenny NS Tracy R Nelson JJ Psaty BM Lipoprotein-associated phospholipase A(2) and risk of congestive heart failure in older adults: the cardiovascular health study. Circ Heart Fail. (2009) 2(5):429–36. 10.1161/CIRCHEARTFAILURE.108.839613 19808373 PMC2756764

[14] Wang X Zhang G Dasgupta S Niewold EL Li C Li Q ATF4 Protects the heart from failure by antagonizing oxidative stress. Circ Res. (2022) 131(1):91–105. 10.1161/CIRCRESAHA.122.321050 35574856 PMC9351829

[15] Ma ZG Yuan YP Zhang X Xu SC Kong CY Song P C1q-tumour necrosis factor-related protein-3 exacerbates cardiac hypertrophy in mice. Cardiovasc Res. (2019) 115(6):1067–77. 10.1093/cvr/cvy279 30407523

[16] Ji YX Zhang P Zhang XJ Zhao YC Deng KQ Jiang X The ubiquitin E3 ligase TRAF6 exacerbates pathological cardiac hypertrophy via TAK1-dependent signalling. Nat Commun. (2016) 7:11267. 10.1038/ncomms11267 27249171 PMC4895385

[17] Guo Y Zhang K Gao X Zhou Z Liu Z Yang K Sustained oligomycin sensitivity conferring protein expression in cardiomyocytes protects against cardiac hypertrophy induced by pressure overload via improving mitochondrial function. Hum Gene Ther. (2020) 31(21-22):1178–89. 10.1089/hum.2020.004 32787458 PMC8024370

[18] Cox EJ Marsh SA. A systematic review of fetal genes as biomarkers of cardiac hypertrophy in rodent models of diabetes. PLOS ONE. (2014) 9(3):e92903. 10.1371/journal.pone.0092903 24663494 PMC3963983

[19] Lopaschuk GD Karwi QG Tian R Wende AR Abel ED. Cardiac energy metabolism in heart failure. Circ Res. (2021) 128(10):1487–513. 10.1161/CIRCRESAHA.121.318241 33983836 PMC8136750

[20] Menendez-Montes I Abdisalaam S Xiao F Lam NT Mukherjee S Szweda LI Mitochondrial fatty acid utilization increases chromatin oxidative stress in cardiomyocytes. Proc Natl Acad Sci U S A. (2021) 118(34):e2101674118. 10.1073/pnas.2101674118 34417314 PMC8403954

[21] Sack MN Rader TA Park S Bastin J McCune SA Kelly DP. Fatty acid oxidation enzyme gene expression is downregulated in the failing heart. Circulation. (1996) 94(11):2837–42. 10.1161/01.cir.94.11.2837 8941110

[22] Porreca E Di Febbo C Mincione G Reale M Baccante G Guglielmi MD Increased transforming growth factor-beta production and gene expression by peripheral blood monocytes of hypertensive patients. Hypertension. (1997) 30(1 Pt 1):134–9. 10.1161/01.hyp.30.1.134 9231833

[23] Flevaris P Khan SS Eren M Schuldt AJT Shah SJ Lee DC Plasminogen activator inhibitor type I controls cardiomyocyte transforming growth factor-*β* and cardiac fibrosis. Circulation. (2017) 136(7):664–79. 10.1161/CIRCULATIONAHA.117.028145 28588076 PMC5784400

[24] Bedi KC Snyder NW Brandimarto J Aziz M Mesaros C Worth AJ Evidence for intramyocardial disruption of lipid metabolism and increased myocardial ketone utilization in advanced human heart failure. Circulation. (2016) 133(8):706–16. 10.1161/CIRCULATIONAHA.115.017545 26819374 PMC4779339

[25] Sun Y Fan W Xue R Dong B Liang Z Chen C Transcribed ultraconserved regions, uc.323, ameliorates cardiac hypertrophy by regulating the transcription of CPT1b (carnitine palmitoyl transferase 1b). Hypertension. (2020) 75(1):79–90. 10.1161/HYPERTENSIONAHA.119.13173 31735087

[26] Legchenko E Chouvarine P Borchert P Fernandez-Gonzalez A Snay E Meier M PPAR*γ* agonist pioglitazone reverses pulmonary hypertension and prevents right heart failure via fatty acid oxidation. Sci Transl Med. (2018) 10(438):eaao0303. 10.1126/scitranslmed.aao0303 29695452

[27] Lian J van der Veen JN Watts R Jacobs RL Lehner R. Carboxylesterase 1d (Ces1d) does not contribute to cholesteryl ester hydrolysis in the liver. J Lipid Res. (2021) 62:100093. 10.1016/j.jlr.2021.100093 34153284 PMC8287225

[28] Li G Li X Yang L Wang S Dai Y Fekry B Adipose tissue-specific ablation of Ces1d causes metabolic dysregulation in mice. Life Sci Alliance. (2022) 5(8):e202101209. 10.26508/lsa.202101209 35459739 PMC9034061

[29] Pike LS Smift AL Croteau NJ Ferrick DA Wu M. Inhibition of fatty acid oxidation by etomoxir impairs NADPH production and increases reactive oxygen species resulting in ATP depletion and cell death in human glioblastoma cells. Biochimica et Biophysica Acta (BBA) - Bioenergetics. (2011) 1807(6):726–34. 10.1016/j.bbabio.2010.10.022 21692241

[30] Dambrova M Zuurbier CJ Borutaite V Liepinsh E Makrecka-Kuka M. Energy substrate metabolism and mitochondrial oxidative stress in cardiac ischemia/reperfusion injury. Free Radic Biol Med. (2021) 165:24–37. 10.1016/j.freeradbiomed.2021.01.036 33484825

[31] Chen Z Jin ZX Cai J Li R Deng KQ Ji YX Energy substrate metabolism and oxidative stress in metabolic cardiomyopathy. J Mol Med (Berl). (2022) 100(12):1721–39. 10.1007/s00109-022-02269-1 36396746

