# Supplementary material for: Identify Tcea3 as a novel anti-cardiomyocyte hypertrophy gene involved in fatty acid oxidation and oxidative stress
Source: Front Cardiovasc Med. 2023 Jun 19;10:1137429. doi: 10.3389/fcvm.2023.1137429 (PMC10315901; doi:10.3389/fcvm.2023.1137429)
Supplement: Supplementary file 1 [file Table1.docx]

# Identify TCEA3 as a novel anti-cardiac hypertrophy gene involved in fatty acid oxidation and oxidative Stress


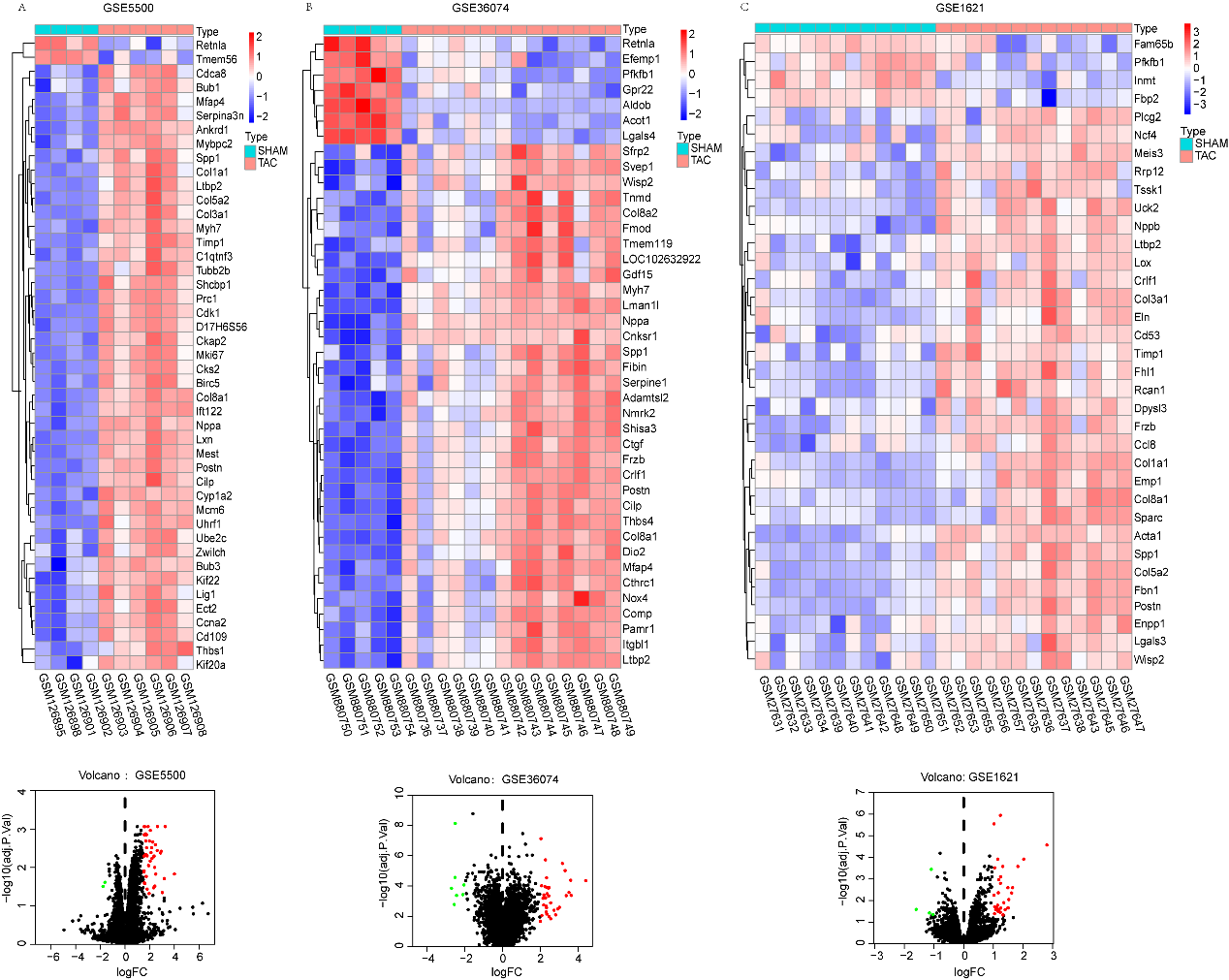


Figure S1 Identification of common DEGs from GSE5500, GSE56348 and GSE1621 datasets. Heat map and volcano plot of GSE5500(A), GSE3674(C) and GSE1621(E) respectively. The t-test was used to analyze DEGs, with the cut-off criteria of |logFC|>0.5 and adj. P<0.05; DEG, differentially expressed gene; GEO, Gene Expression Omnibus; logFC, log-fold change.


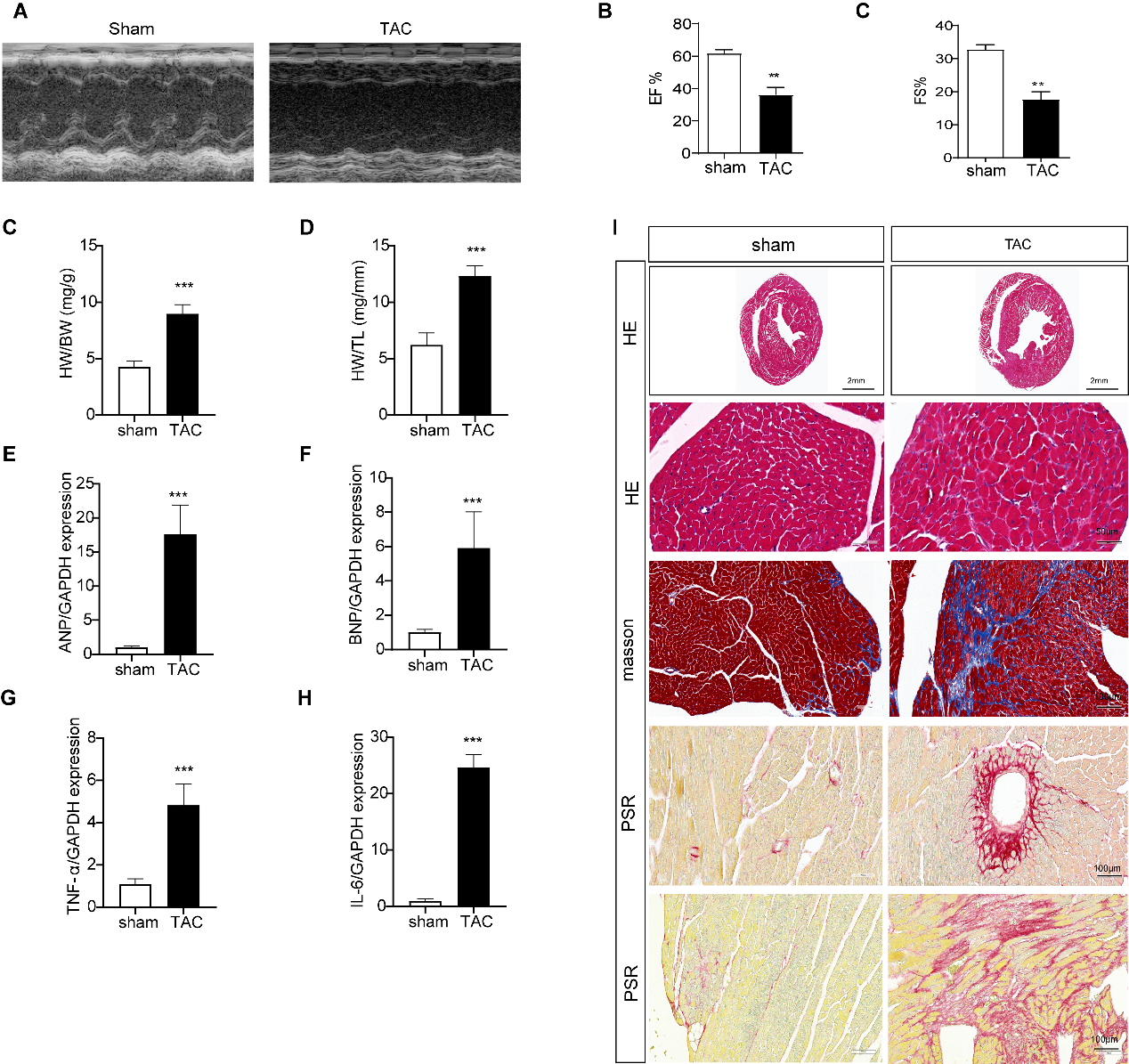


Figure S2 Cardiac insufficiency in mice after TAC surgery. (A-C) represent the ejection fraction (EF%); systolic short-axis shortening (FS%) in mice after sham (Sham) and TAC surgery ultrasound, respectively. (C-D) Heart weight(HW)/body weight (BW) and HW/tibia length (TL) ratios after sham and TAC surgery, respectively. (E-F) represent the mRNA levels of ANP and BNP in mice after sham and TAC surgery, respectively. (G-H) represent the mRNA levels of TNF-α and IL-6 in mice after sham and TAC surgery, respectively. (I) Representative histological examinations of the hearts of two groups of mice after sham (Sham) and TAC surgery, respectively. HE staining reflects the size of cardiomyocytes and Masson and Sirius Red staining (PSR) reflects the degree of myocardial fibrosis. *p < 0.05, **P < 0.01, ***P < 0.001 compared with sham group, data are presented as mean±SEM The data are presented as mean±SEM.


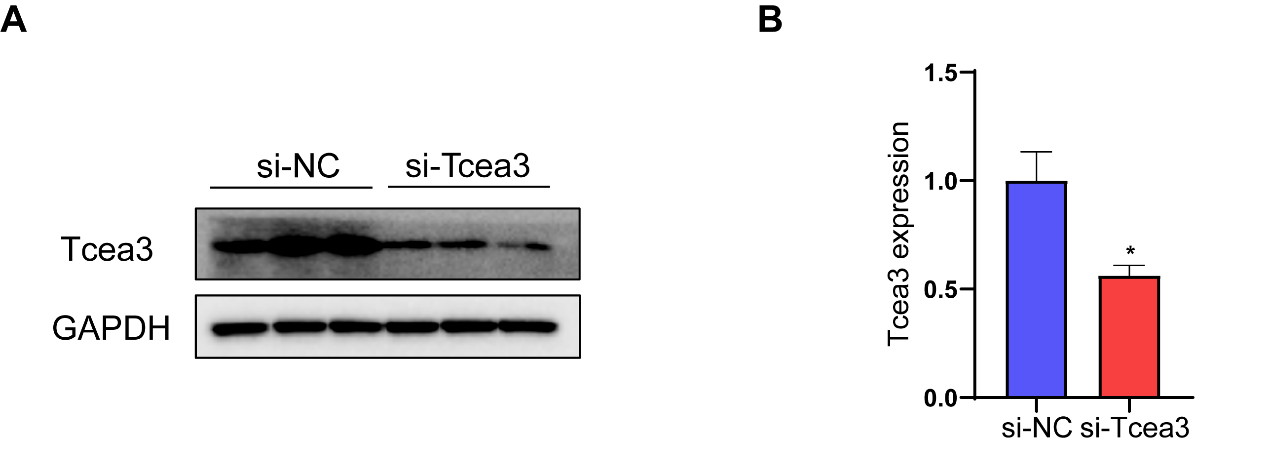


Figure S3 (A,B) Western blot and quantitation of Tcea3 protein level in NRVMs transfected with control siRNA or Tcea3 siRNA(n=3). Data are presented as mean ± SEM. *p < 0.05.

Table SI. The common DEGs of three gene expression profiles (P <0.05, |logFC|>0.5).

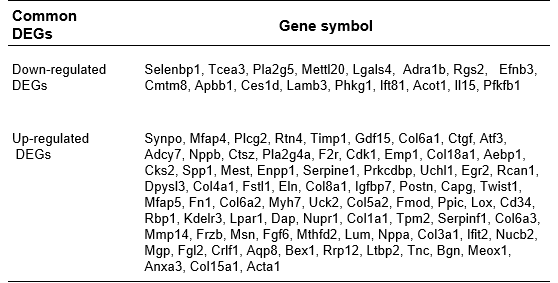


[Table S2](https://www.ncbi.nlm.nih.gov/pmc/articles/PMC6908929/figure/f2-or-0-0-7400/). Significant enriched analysis of differentially expressed genes


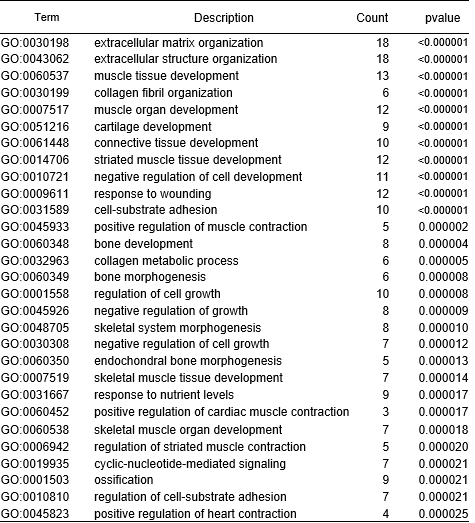


[Table S3](https://www.ncbi.nlm.nih.gov/pmc/articles/PMC6908929/figure/f2-or-0-0-7400/) Correlation analysis between DEGs and heart failure progression


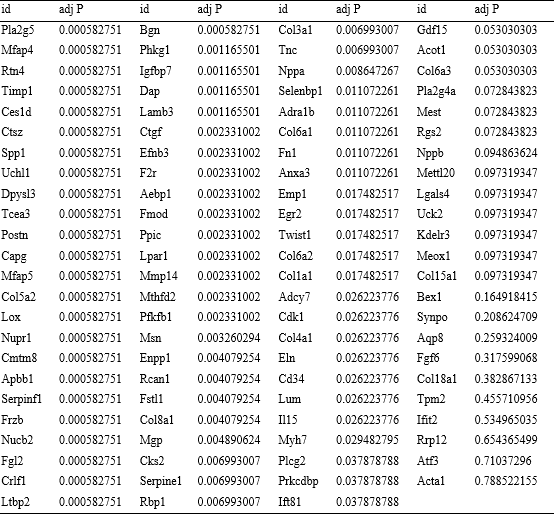


Table S4 The demographics of human heart samples

| Subject | Diagnosis | Age(years) | Gender | LVEF(%) | LVEDd(mm) | IVSd(mm) |
| --- | --- | --- | --- | --- | --- | --- |
| 1 | Donor | 57 | Female | 62 | 39 | 7 |
| 2 | Donor | 50 | Female | N/A | N/A | N/A |
| 3 | Donor | 28 | Male | 72 | 45 | 7 |
| 4 | Donor | 53 | Male | 65 | 47 | 8 |
| 5 | Donor | 45 | Male | 66 | 43 | 9 |
| 6 | Donor | 53 | Male | 63 | 46 | 9 |
|  |  |  |  |  |  |  |
| 7 | DCM | 63 | Male | 23 | 70 | 11 |
| 8 | DCM | 39 | Female | 26 | 60 | 9 |
| 9 | DCM | 38 | Male | 30 | 67 | 8 |
| 10 | DCM | 44 | Female | 22 | 62 | 10 |
| 11 | DCM | 56 | Male | 23 | 90 | 10 |
| 12 | DCM | 64 | Male | 37 | 80 | 10 |
| 13 | DCM | 76 | Male | 36 | 60 | 9 |

DCM: Dilated cardiomyopathy; LVEF: Left ventricular ejection fraction; LVEDd: Left ventricular end-diastolic diameter; IVSd: Interventricular septal thickness at diastole; N/A: not available.

Table S5 Primers for qPCR

| **Gene** |  | **Sequence (5’-3’)** |
| --- | --- | --- |
| Mouse Tcea3 | Forward | AGCAGCTCTGAAGGCAGAAG |
|  | Reverse | CTCATCACTGGCCATTTCCT |
| Rat Tcea3 | Forward | CATCCAGCTGCTACAGACCA |
|  | Reverse | CTGGTTTCCAGCCTGAAGAG |
| Mouse GAPDH | Forward | AGGTCGGTGTGAACGGATTTG |
|  | Reverse | TGTAGACCATGTAGTTGAGGTCA |
| Rat GAPDH | Forward | ACAGCAACAGGGTGGTGGAC |
|  | Reverse | TTTGAGGGTGCAGCGAACTT |
| Rat ANP | Forward | ATACAGTGCGGTGTCCAACA |
|  | Reverse | AGCCCTCAGTTTGCTTTTCA |
| Rat BNP | Forward | CAGCTCTCAAAGGACCAAGG |
|  | Reverse | GCAGCTTGAACTATGTGCCA |
| Rat Myh6 | Forward | ACTCATGGCCACACTCTTCT |
|  | Reverse | AAGTGAGGATGGGTGGTCCT |
| Rat Myh7 | Forward | GCTCCTAAGTAATCTGTTTG |
|  | Reverse | AAGTGAGGGTGCGTGGAGCG |
| Rat Pla2g5 | Forward | AACTGAGGCTCTGACTCCATTC |
|  | Reverse | CTCATCTTCCAGAACTGATATG |
| Rat Ces1d | Forward | AGA TTC AGT GAC CGT CTT TGG |
|  | Reverse | TGG CCA GAG GAG ATA AGA CAA |
| Rat Pla2g4a | Forward | CTTGCATTCTACACGTGATGTGCC |
|  | Reverse | GATGTATTGAGATTCAAGCCCAGC |
| Rat Plcg2 | Forward | TCAATGGGCGGACAGGTTA |
|  | Reverse | CTAGTTTAGGGAGGTGGCGTG |
| Rat CPT1B | Forward | GTC GCT TCT TCA AGGTTT GG |
|  | Reverse | AAG AAA GCA GCA CGTTCG AT |
| Rat LCAD | Forward | CCCTGGTTTCAGCCTCCATT |
|  | Reverse | TCACTCCCAGACCTTTTGGC |
| Rat MCAD | Forward | AGCCTTCACCGGATTCATCG |
|  | Reverse | AGCCCCCATTGCAATCTTGA |
